# Supplementary material for: Healthcare resource utilisation and cost of pneumococcal disease from 2003 to 2019 in children ≤17 years in England
Source: PLoS One. 2023 Apr 5;18(4):e0283084. doi: 10.1371/journal.pone.0283084 (PMC10075442; doi:10.1371/journal.pone.0283084)
Supplement: S1 File — (DOCX) [file pone.0283084.s001.docx]

# Supporting information, S1-S12 Tables

**S1 Table. IPD, PP, ACP and AOM diagnosis code lists**

| **Disease** | **Read diagnosis codes** | **ICD-10 diagnosis codes** |
| --- | --- | --- |
| **IPD** | - | A40.3, A40.9+B95.3, G00.1, G00.2+B95.3, J13+A40.3, J13+A40.9, J13+A41.9, J13+A49.9, J86.0+B95.3, J86.9+B95.3, J85.1+B95.3, I30.1+B95.3, I33.0+B95.3, I33.9+B95.3, K65.0+B95.3, K65.8+B95.3, K65.9+B95.3, M00.1, M86.1+B95.3, M86.2+B95.3, M86.9+B95.3 |
| **PP** | AyuK300, A3BX400, H223.00, H21..00, H21..11 | J13 |
| **ACP** | 14B2.00, 43eG.00, 43eH.00, 43n1.00, 43n7.00, 4JRC.00, 4JUK.00, A022200, A116.00, A203.00, A204.00, A205.00, A3A4000, A3BXA00, A3BXB00, A3By400, A521.00, A54x400, A551.00, A730.00, A785000, A789300, A789311, A789900, AB24.11, AB40500, AB41500, AD04.00, AyuK900, AyuKA00, H060A00, H2...00, H20..00, H20..11, H200.00, H201.00, H202.00, H203.00, H20y.00, H20z.00, H21..00, H21..11, H22..00, H22..11, H220.00, H221.00, H222.00, H222.11, H223.00, H223000, H224.00, H22y.00, H22y000, H22y011, H22y100, H22y200, H22yX00, H22yz00, H22z.00, H23..00, H23..11, H230.00, H231.00, H232.00, H233.00, H23z.00, H24..00, H240.00, H241.00, H242.00, H243.00, H243.11, H244.00, H246.00, H247.00, H247000, H247100, H247z00, H24y.00, H24y000, H24y100, H24y200, H24y300, H24y400, H24y500, H24y600, H24y700, H24yz00, H24z.00, H25..00, H25..11; H26..00; H260.00; H261.00; H262.00; H263.00; H270.00; H270.11; H270000; H270100; H270z00; H28..00; H2B..00; H2C..00; H2y..00; H2z..00; H35..11; H357.00; H35z.00; H35z100; H35zz00; H460.00; H460100; H460z00; H47..00; H47..11; H470.00; H470.11; H470000; H470100; H470200; H470211; H470300; H470311; H470312; H470z00; H471.00; H471000; H471z00; H472.00; H47y.00; H47yz00; H47z.00; H4y0000; H530200; H530300; H540000; H540100; H563300; H564.00; H564.11; H56y000; H56y100; H571.00; Hyu0800; Hyu0A00; Hyu0B00; Hyu0C00; Hyu0D00; Hyu0G00; Hyu0H00; Hyu4300; Hyu4700; Q310.00; Q310000; Q310100; Q310200; Q310300; Q310400; Q310500; Q310600; Q310y00; Q310z00; Q311z11; SP13100; AyuK300; A3BX400 | A22.1, A37.9, A48.1, B25.0,B44.0, J10.0, J11.0, J12.0, J12.1, J12.2, J12.3, J12.8, J12.9, J13, J14, J15.0, J15.1, J15.2, J15.3, J15.4, J15.5, J15.6, J15.7, J15.8, J15.9, J16.0, J16.8, J17.0, J17.1, J17.2, J17.3, J17.8, J18.0, J18.1, J18.2, J18.8, J18.9 |
| **AOM** | F526.00; F528.00; F527.00; F525.00; SN30.11; A552.00; F520100; F520z00; F520.00; F520300; F520000; F52..00; F524000; F523.00; FyuP200; F52z.00; FyuP400; FyuP300; F521.00; F522.00 | - |

ACP: all-cause pneumonia; AOM: acute otitis media; ICD-10: International Statistical Classification of Diseases and Related Health Problems 10^th^ revision; IPD: invasive pneumococcal disease; PP: pneumococcal pneumonia.

**S2 Table. Healthcare resource reference units**

| **Resource** | **Unit** | | **Source of reference cost** | **Reference Year** | **Cost** |
| --- | --- | --- | --- | --- | --- |
| **General practitioner** | | |  |  |  |
| GP visit | GP with qualification costs: Per surgery consultation lasting 9.22 minutes, including direct care staff costs | | Curtis, Lesley A. and Burns, Amanda (2020) Unit Costs of Health & Social Care 2020. Unit Costs of Health and Social Care. PSSRU, University of Kent, 185 pp. ISBN 978-1-911353-12-6. [46] | 2019/20 | £39.00 |
| GP antibiotic prescription | Assessed for each antibiotic based on the available literature, guidance and SmPC^a^ | | English Prescribing Dataset (EPD) 2019 [45] | 2019/20 | - |
| **Inpatient** |  | |  |  |  |
| Hospital Admission | Median cost of hospitalisations of the type described in each row in children 0-17 years during 2003-2019 obtained after the application of the HRG tariff to the episodes corresponding to each hospitalisation identified in HES | | HES HRG + NHS Trust Reference Cost Schedules for years 2003-2012^b^ / Secondary Uses Services (SUS) Guidance tariff reference data for years 2013-2019 (https://digital.nhs.uk/binaries/content/assets/website-assets/services/sus/sus-guidance/sus-tariff-reference-data-v1.4.xlsx) [47] | 2019/20 Tariffs have been updated to 2019/2020 using the inflation calculator from the Bank of England (https://www.bankofengland.co.uk/monetary-policy/inflation/inflation-calculator) [48] |  |
| Pneumonia | Hospitalisation for all cause pneumonia. Correspond to a median hospital stay of 3 days | |  |  | £4,443.80 |
| IPD | Hospitalisation for IPD. Correspond to a median hospital stay of 6 days | |  |  | £13,062.00 |
| Bacteraemia / Septicaemia | Hospitalisation for pneumococcal bacteraemia / septicaemia. Correspond to a median hospital stay of 3 days | |  |  | £4,020.50 |
| Meningitis | Hospitalisation for pneumococcal meningitis. Correspond to a median hospital stay of 5 days | |  |  | £7,886.00 |
| Bacteraemic pneumonia | Hospitalisation for pneumococcal bacteraemic pneumonia. Correspond to a median hospital stay of 8 days | |  |  | £28,661.40 |
| Other IPD | Hospitalisation for other pneumococcal invasive diseases Correspond to a median hospital stay of 9 days | |  |  | £13,574.80 |
| Day in Hospital | | |  |  |  |
| ACP | | |  |  | £1,253.95 |
| IPD | | |  |  | £2,615.00 |
| Bacteraemia/Septicemia | | |  |  | £2,069.50 |
| Meningitis |  | |  |  | £1,951.00 |
| Bacteraemic pneumonia | |  |  |  | £4,070.36 |
| Other IPD |  | |  |  | £1,338.35 |

AOM: acute otitis media; GP: general practice; IPD: invasive pneumococcal disease; SmPC: summary of product characteristics.

^a^NICE Guidelines Otitis media (acute): antimicrobial prescribing [43], NICE Pneumonia (community-acquired): antimicrobial prescribing [44] and SmPC information captured from https://www.medicines.org.uk/emc

^b^References for NHS Trust Reference Cost Schedules for years 2003-2012:

https://webarchive.nationalarchives.gov.uk/ukgwa/20100604202759/http://www.dh.gov.uk/en/Publicationsandstatistics/Publications/PublicationsPolicyAndGuidance/DH_4105545 // file: dh_4105553.xls

https://webarchive.nationalarchives.gov.uk/ukgwa/20100509080731/http://dh.gov.uk/en/Publicationsandstatistics/Publications/PublicationsPolicyAndGuidance/DH_4133221 // file:dh_4133225.xls

https://webarchive.nationalarchives.gov.uk/ukgwa/20100509080731/http://dh.gov.uk/en/Publicationsandstatistics/Publications/PublicationsPolicyAndGuidance/DH_062884 //file: dh_062882.xls

https://webarchive.nationalarchives.gov.uk/ukgwa/20110503153250/http://www.dh.gov.uk/en/Publicationsandstatistics/Publications/PublicationsPolicyAndGuidance/DH_082571 //file: dh_118333.xls

https://webarchive.nationalarchives.gov.uk/ukgwa/20110503153249/http://www.dh.gov.uk/en/Publicationsandstatistics/Publications/PublicationsPolicyAndGuidance/DH_098945 //file: dh_098948.xls

https://webarchive.nationalarchives.gov.uk/ukgwa/20110503152316/http://www.dh.gov.uk/en/Publicationsandstatistics/Publications/PublicationsPolicyAndGuidance/DH_111591 //file:dh_118322.xls

https://webarchive.nationalarchives.gov.uk/ukgwa/20110503153248/http://www.dh.gov.uk/en/Publicationsandstatistics/Publications/PublicationsPolicyAndGuidance/DH_123459 //file:dh_123455.xls

https://www.gov.uk/government/publications/2010-11-reference-costs-publication //file:dh_131145.xls

https://www.gov.uk/government/publications/nhs-reference-costs-financial-year-2011-to-2012 //file: NSRC01-2011-12.xls

https://www.gov.uk/government/publications/confirmation-of-payment-by-results-pbr-arrangements-for-2012-13 // file dh_133578.xls

**S3 Table. IPD HCRU by study year**

|  | **All children** | | | **<2 years** | | | **2-4 years** | | | **5-17 years** | | |
| --- | --- | --- | --- | --- | --- | --- | --- | --- | --- | --- | --- | --- |
|  | **N of episodes** | **N of hospital admissions** | **Inpatient admission yearly rate per 1,000 patients (95% CI)** | **N of episodes** | **N of hospital admissions** | **Inpatient admission yearly rate per 1,000 patients (95% CI)** | **N of episodes** | **N of hospital admissions** | **Inpatient admission yearly rate per 1,000 patients (95% CI)** | **N of episodes** | **N of hospital admissions** | **Inpatient admission yearly rate per 1,000 patients (95% CI)** |
| **2003** | 14 | 23 | 0.05 (0.03-0.07) | 8 | 14 | 0.39 (0.21-0.65) | 5 | 8 | 0.10 (0.04-0.20) | <5 | <5 | NR^a^ |
| **2004** | 16 | 24 | 0.05 (0.03-0.07) | 14 | 21 | 0.49 (0.30-0.75) | <5 | <5 | NR^a^ | <5 | <5 | NR^a^ |
| **2005** | 19 | 31 | 0.06 (0.04-0.08) | 10 | 20 | 0.44 (0.27-0.68) | 5 | 6 | 0.07 (0.03-0.16) | <5 | 5 | 0.01 (0.00-0.03) |
| **2006** | 21 | 33 | 0.06 (0.04-0.09) | 14 | 24 | 0.51 (0.33-0.76) | <5 | 5 | 0.06 (0.02-0.13) | <5 | <5 | NR^a^ |
| **2007** | 15 | 26 | 0.05 (0.03-0.07) | 7 | 15 | 0.30 (0.17-0.50) | <5 | <5 | NR^a^ | <5 | 7 | 0.02 (0.01-0.04) |
| **2008** | 17 | 34 | 0.06 (0.04-0.08) | 13 | 29 | 0.57 (0.38-0.81) | <5 | <5 | NR^a^ | <5 | <5 | NR^a^ |
| **2009** | 8 | 13 | 0.02 (0.01-0.04) | <5 | 9 | 0.18 (0.08-0.33) | <5 | <5 | NR^a^ | <5 | <5 | NR^a^ |
| **2010** | 10 | 13 | 0.02 (0.01-0.04) | 6 | 7 | 0.14 (0.06-0.28) | <5 | <5 | NR^a^ | <5 | <5 | NR^a^ |
| **2011** | 10 | 16 | 0.03 (0.02-0.05) | <5 | 10 | 0.20 (0.10-0.36) | <5 | <5 | NR^a^ | <5 | <5 | NR^a^ |
| **2012** | 16 | 28 | 0.05 (0.03-0.08) | 8 | 17 | 0.34 (0.20-0.55) | 6 | 8 | 0.08 (0.04-0.17) | <5 | <5 | NR^a^ |
| **2013** | <5 | 6 | 0.01 (0.00-0.03) | <5 | <5 | NR^a^ | <5 | <5 | NR^a^ | <5 | <5 | NR^a^ |
| **2014** | <5 | <5 | NR^a^ | <5 | <5 | NR^a^ | <5 | <5 | NR^a^ | <5 | <5 | NR^a^ |
| **2015** | 9 | 13 | 0.04 (0.02-0.06) | 6 | 10 | 0.34 (0.16-0.63) | <5 | <5 | NR^a^ | <5 | <5 | NR^a^ |
| **2016** | <5 | <5 | NR^a^ | <5 | <5 | NR^a^ | 0 | 0 | 0.00 (0.00-0.08) | 0 | 0 | 0.00 (0.00-0.02) |
| **2017** | <5 | <5 | NR^a^ | <5 | <5 | NR^a^ | 0 | 0 | 0.00 (0.00-0.11) | 0 | 0 | 0.00 (0.00-0.02) |
| **2018** | <5 | <5 | NR^a^ | 0 | 0 | 0.00 (0.00-0.27) | 0 | 0 | 0.00 (0.00-0.13) | <5 | <5 | NR^a^ |
| **2019** | <5 | 5 | 0.03 (0.01-0.08) | <5 | <5 | NR^a^ | <5 | <5 | NR^a^ | <5 | <5 | NR^a^ |
| **Trend test**  **(p-value)** | - | - | 0.079 | - | - | 0.099 | - | - | NP^b^ | - | - | NP^b^ |

^a^Not reported: Where the number of hospital admissions were less than 5 the data are not reported, in accordance with CPRD and HES-APC data protection policies. ^b^Not performed: Not enough available data to perform the Mann Kendall Test. CI: confidence interval; IPD: invasive pneumococcal disease; HCRU: healthcare resource utilisation; N: number.

**S4 Table. IPD costs per episode by study year**

|  | **Inpatient cost per episode, £ (95% CI)** | | | |
| --- | --- | --- | --- | --- |
|  | **All children** | **<2 years** | **2-4 years** | **5-17 years** |
| **2003** | 48,176 (25,741-70,612) | 32,688 (17,814-47,561) | 88,583 (27,278-149,888) | NR^a^ |
| **2004** | 25,660 (14,984-36,335) | 27,644 (15,686-39,602) | NR^a^ | NR^a^ |
| **2005** | 21,883 (16,135-27,632) | 23,274 (14,980-31,567) | 23,012 (8,239-37,785) | NR^a^ |
| **2006** | 28,267 (15,577-40,957) | 34,182 (15,629-52,735) | NR^a^ | NR^a^ |
| **2007** | 37,357 (16,685-58,029) | 46,696 (7,440-85,953) | NR^a^ | NR^a^ |
| **2008** | 31,226 (18,352-44,100) | 32,185 (15,209-49,160) | NR^a^ | NR^a^ |
| **2009** | 23,862 (7,031-40,692) | NR^a^ | NR^a^ | NR^a^ |
| **2010** | 28,242 (14,586-41,898) | 33,123 (9,859-56,388) | NR^a^ | NR^a^ |
| **2011** | 29,288 (18,459-40,117) | NR^a^ | NR^a^ | NR^a^ |
| **2012** | 46,253 (10,326-82,180) | 32,361 (6,840-57,881) | 40,097 (0-108,487) | NR^a^ |
| **2013** | NR^a^ | NR^a^ | NR^a^ | NR^a^ |
| **2014** | NR^a^ | NR^a^ | NR^a^ | NR^a^ |
| **2015** | 46,198 (14,832-77,564) | 61,888 (17,718-106,059) | NR^a^ | NR^a^ |
| **2016** | NR^a^ | NR^a^ | NR^a^ | NR^a^ |
| **2017** | NR^a^ | NR^a^ | NR^a^ | NR^a^ |
| **2018** | NR^a^ | NR^a^ | NR^a^ | NR^a^ |
| **2019** | NR^a^ | NR^a^ | NR^a^ | NR^a^ |
| **Trend test**  **(p-value)** | 0.436 | 0.251 | NP^b^ | NP^b^ |

^a^Not reported: Where the number of episodes were less than 5 the data are not reported, in accordance with CPRD and HES-APC data protection policies. ^b^Not performed: Not enough available data to perform the Mann Kendall Test. CI: confidence interval; IPD: invasive pneumococcal disease; N: number.

**S5 Table. PP HCRU in primary care by study year**

|  | **All children** | | | **<2 years** | | | **2-4 years** | | | **5-17 years** | | |
| --- | --- | --- | --- | --- | --- | --- | --- | --- | --- | --- | --- | --- |
|  | **N of episodes** | **N of GP visits** | **GP visits yearly rate per 1,000 patients**  **(95% CI)** | **N of episodes** | **N of GP visits** | **GP visits yearly rate per 1,000 patients**  **(95% CI)** | **N of episodes** | **N of GP visits** | **GP visits yearly rate per 1,000 patients**  **(95% CI)** | **N of episodes** | **N of GP visits** | **GP visits yearly rate per 1,000 patients**  **(95% CI)** |
| **2003** | 63 | 64 | 0.14  (0.10-0.17) | 15 | 15 | 0.42  (0.23-0.69) | 27 | 28 | 0.36  (0.24-0.52) | 21 | 21 | 0.06  (0.04-0.09) |
| **2004** | 70 | 65 | 0.13  (0.10-0.17) | 19 | 16 | 0.37  (0.21-0.61) | 28 | 27 | 0.34  (0.22-0.49) | 23 | 22 | 0.06  (0.04-0.09) |
| **2005** | 86 | 80 | 0.15  (0.12-0.19) | 19 | 16 | 0.35  (0.20-0.57) | 33 | 31 | 0.37  (0.25-0.52) | 34 | 33 | 0.08  (0.06-0.12) |
| **2006** | 95 | 90 | 0.17  (0.13-0.21) | 23 | 21 | 0.45  (0.28-0.68) | 32 | 29 | 0.33  (0.22-0.47) | 40 | 40 | 0.10  (0.07-0.14) |
| **2007** | 78 | 73 | 0.13  (0.10-0.17) | 18 | 15 | 0.30  (0.17-0.50) | 19 | 22 | 0.24  (0.15-0.36) | 41 | 36 | 0.09  (0.06-0.12) |
| **2008** | 65 | 53 | 0.09  (0.07-0.12) | 19 | 17 | 0.33  (0.19-0.53) | 21 | 15 | 0.16  (0.09-0.26) | 25 | 21 | 0.05  (0.03-0.08) |
| **2009** | 61 | 53 | 0.09  (0.07-0.12) | 18 | 15 | 0.29  (0.16-0.48) | 18 | 15 | 0.16  (0.09-0.26) | 25 | 23 | 0.06  (0.03-0.08) |
| **2010** | 56 | 47 | 0.08  (0.06-0.11) | 8 | 8 | 0.16  (0.07-0.31) | 22 | 20 | 0.20  (0.13-0.32) | 26 | 19 | 0.05  (0.03-0.07) |
| **2011** | 53 | 53 | 0.10  (0.07-0.13) | 18 | 17 | 0.34  (0.20-0.54) | 11 | 12 | 0.13  (0.06-0.22) | 24 | 24 | 0.06  (0.04-0.09) |
| **2012** | 37 | 36 | 0.07  (0.05-0.09) | 9 | 7 | 0.14  (0.06-0.29) | 13 | 14 | 0.15  (0.08-0.25) | 15 | 15 | 0.04  (0.02-0.06) |
| **2013** | 33 | 27 | 0.05  (0.04-0.08) | 6 | 5 | 0.11  (0.04-0.25) | 15 | 14 | 0.16  (0.09-0.26) | 12 | 8 | 0.02  (0.01-0.04) |
| **2014** | 28 | 23 | 0.05  (0.03-0.08) | 11 | 8 | 0.21  (0.09-0.42) | 7 | 6 | 0.08  (0.03-0.17) | 10 | 9 | 0.03  (0.01-0.05) |
| **2015** | 15 | 14 | 0.04  (0.02-0.07) | <5 | <5 | NR^a^ | 9 | 9 | 0.15  (0.07-0.28) | <5 | <5 | NR^a^ |
| **2016** | 11 | 13 | 0.05  (0.03-0.09) | <5 | <5 | NR^a^ | 5 | 5 | 0.11  (0.04-0.27) | 7 | 7 | 0.04  (0.01-0.08) |
| **2017** | 8 | 8 | 0.04  (0.02-0.08) | <5 | <5 | NR^a^ | <5 | <5 | NR^a^ | <5 | <5 | NR^a^ |
| **2018** | 7 | 5 | 0.03  (0.01-0.07) | <5 | <5 | NR^a^ | <5 | <5 | NR^a^ | 0 | 0 | 0.00  (0.00-0.03) |
| **2019** | <5 | <5 | NR^a^ | <5 | <5 | NR^a^ | 0 | 0 | 0.00  (0.00-0.15) | <5 | <5 | NR^a^ |
| **Trend test**  **(p-value)** | - | - | <0.001 | - | - | 0.003 | - | - | <0.001 | - | - | 0.003 |

^a^Not reported: Where the number of GP visits were less than 5 the data are not reported, in accordance with CPRD and HES-APC data protection policies. CI: confidence interval; GP: general practice; HCRU: healthcare resource utilisation; N: number; PP: pneumococcal pneumonia.

**S6 Table. ACP HCRU in primary care by study year**

|  | **All children** | | | **<2 years** | | | **2-4 years** | | | **5-17 years** | | |
| --- | --- | --- | --- | --- | --- | --- | --- | --- | --- | --- | --- | --- |
|  | **N of episodes** | **N of GP visits** | **GP visits yearly rate per 1,000 patients**  **(95% CI)** | **N of episodes** | **N of GP visits** | **GP visits yearly rate per 1,000 patients**  **(95% CI)** | **N of episodes** | **N of GP visits** | **GP visits yearly rate per 1,000 patients**  **(95% CI)** | **N of episodes** | **N of GP visits** | **GP visits yearly rate per 1,000 patients**  **(95% CI)** |
| **2003** | 719 | 570 | 1.21  (1.11-1.31) | 216 | 161 | 4.47  (3.81-5.22) | 279 | 205 | 2.65  (2.30-3.04) | 224 | 204 | 0.57  (0.49-0.65) |
| **2004** | 836 | 630 | 1.26  (1.16-1.36) | 288 | 191 | 4.47  (3.85-5.15) | 306 | 241 | 2.99  (2.63-3.40) | 242 | 198 | 0.52  (0.45-0.60) |
| **2005** | 945 | 741 | 1.42  (1.32-1.52) | 296 | 205 | 4.51  (3.92-5.18) | 326 | 265 | 3.15  (2.78-3.55) | 323 | 271 | 0.69  (0.61-0.78) |
| **2006** | 960 | 753 | 1.40  (1.30-1.50) | 273 | 187 | 3.99  (3.44-4.60) | 339 | 264 | 3.00  (2.65-3.39) | 348 | 302 | 0.75  (0.67-0.84) |
| **2007** | 894 | 649 | 1.18  (1.09-1.27) | 266 | 155 | 3.14  (2.66-3.67) | 334 | 253 | 2.77  (2.44-3.13) | 294 | 241 | 0.59  (0.52-0.67) |
| **2008** | 872 | 592 | 1.05  (0.97-1.14) | 292 | 166 | 3.24  (2.77-3.77) | 299 | 210 | 2.21  (1.92-2.53) | 281 | 216 | 0.52  (0.45-0.59) |
| **2009** | 1,487 | 1,218 | 2.15  (2.04-2.28) | 341 | 225 | 4.38  (3.82-4.99) | 439 | 358 | 3.70  (3.33-4.11) | 707 | 635 | 1.52  (1.41-1.64) |
| **2010** | 968 | 618 | 1.11  (1.02-1.20) | 286 | 165 | 3.25  (2.77-3.78) | 378 | 244 | 2.50  (2.20-2.83) | 304 | 209 | 0.51  (0.44-0.58) |
| **2011** | 920 | 586 | 1.07  (0.99-1.16) | 275 | 160 | 3.17  (2.70-3.70) | 340 | 205 | 2.14  (1.86-2.46) | 305 | 221 | 0.55  (0.48-0.63) |
| **2012** | 884 | 511 | 0.95  (0.87-1.03) | 256 | 126 | 2.53  (2.11-3.01) | 328 | 195 | 2.05  (1.77-2.36) | 300 | 190 | 0.48  (0.42-0.56) |
| **2013** | 656 | 404 | 0.80  (0.72-0.88) | 212 | 120 | 2.62  (2.17-3.14) | 242 | 143 | 1.61  (1.36-1.90) | 202 | 141 | 0.38  (0.32-0.45) |
| **2014** | 581 | 319 | 0.73  (0.65-0.81) | 174 | 99 | 2.64  (2.14-3.21) | 204 | 106 | 1.39  (1.13-1.68) | 203 | 114 | 0.35  (0.29-0.42) |
| **2015** | 418 | 243 | 0.69  (0.61-0.78) | 130 | 80 | 2.72  (2.16-3.39) | 157 | 92 | 1.52  (1.22-1.86) | 131 | 71 | 0.27  (0.21-0.34) |
| **2016** | 372 | 182 | 0.71  (0.61-0.82) | 99 | 47 | 2.20  (1.62-2.93) | 139 | 64 | 1.47  (1.13-1.88) | 134 | 71 | 0.37  (0.29-0.47) |
| **2017** | 236 | 122 | 0.59  (0.49-0.71) | 71 | 33 | 1.90  (1.31-2.67) | 83 | 42 | 1.23  (0.89-1.66) | 82 | 47 | 0.30  (0.22-0.40) |
| **2018** | 219 | 96 | 0.55  (0.45-0.67) | 62 | 25 | 1.81  (1.17-2.67) | 92 | 32 | 1.11  (0.76-1.57) | 65 | 39 | 0.30  (0.21-0.40) |
| **2019** | 175 | 77 | 0.52  (0.41-0.65) | 34 | 13 | 1.14  (0.61-1.95) | 73 | 26 | 1.09  (0.71-1.60) | 68 | 38 | 0.34  (0.24-0.46) |
| **Trend test**  **(p-value)** | - | - | <0.001 | - | - | <0.001 | - | - | <0.001 | - | - | <0.001 |

ACP: all-cause pneumonia; CI: confidence interval; GP: general practice; HCRU: healthcare resource utilisation; N: number.

**S7 Table. PP HCRU in hospital setting by study year**

|  | **All children** | | | **<2 years** | | | **2-4 years** | | | **5-17 years** | | |
| --- | --- | --- | --- | --- | --- | --- | --- | --- | --- | --- | --- | --- |
|  | **N of episodes** | **N of hospital admissions** | **Inpatient admission yearly rate per 1,000 patients**  **(95% CI)** | **N of episodes** | **N of hospital admissions** | **Inpatient admission yearly rate per 1,000 patients**  **(95% CI)** | **N of episodes** | **N of hospital admissions** | **Inpatient admission yearly rate per 1,000 patients**  **(95% CI)** | **N of episodes** | **N of hospital admissions** | **Inpatient admission yearly rate per 1,000 patients (95% CI)** |
| **2003** | 63 | <5 | NR^a^ | 15 | <5 | NR^a^ | 27 | <5 | NR^a^ | 21 | <5 | NR^a^ |
| **2004** | 70 | 12 | 0.02  (0.01-0.04) | 19 | 6 | 0.14  (0.05-0.31) | 28 | <5 | NR^a^ | 23 | <5 | NR^a^ |
| **2005** | 86 | 17 | 0.03  (0.02-0.05) | 19 | 5 | 0.11  (0.04-0.26) | 33 | 8 | 0.10  (0.04-0.19) | 34 | <5 | NR^a^ |
| **2006** | 95 | 23 | 0.04  (0.03-0.06) | 23 | 9 | 0.19  (0.09-0.36) | 32 | 7 | 0.08  (0.03-0.16) | 40 | 7 | 0.02  (0.01-0.04) |
| **2007** | 78 | 14 | 0.03  (0.01-0.04) | 18 | <5 | NR^a^ | 19 | <5 | NR^a^ | 41 | 9 | 0.02  (0.01-0.04) |
| **2008** | 65 | 18 | 0.03  (0.02-0.05) | 19 | <5 | NR^a^ | 21 | 7 | 0.07  (0.03-0.15) | 25 | 7 | 0.02  (0.01-0.03) |
| **2009** | 61 | 17 | 0.03  (0.02-0.05) | 18 | <5 | NR^a^ | 18 | <5 | NR^a^ | 25 | 9 | 0.02  (0.01-0.04) |
| **2010** | 56 | 15 | 0.03  (0.02-0.04) | 8 | <5 | NR^a^ | 22 | 5 | 0.05  (0.02-0.12) | 26 | 9 | 0.02  (0.01-0.04) |
| **2011** | 53 | <5 | NR^a^ | 18 | <5 | NR^a^ | 11 | <5 | NR^a^ | 24 | <5 | NR^a^ |
| **2012** | 37 | <5 | NR^a^ | 9 | <5 | NR^a^ | 13 | 0 | 0.00  (0.00-0.04) | 15 | <5 | NR^a^ |
| **2013** | 33 | 11 | 0.02  (0.01-0.04) | 6 | <5 | NR^a^ | 15 | <5 | NR^a^ | 12 | 8 | 0.02  (0.01-0.04) |
| **2014** | 28 | 6 | 0.01  (0.01-0.03) | 11 | <5 | NR^a^ | 7 | <5 | NR^a^ | 10 | <5 | NR^a^ |
| **2015** | 15 | <5 | NR^a^ | <5 | 0 | 0.00  (0.00-0.13) | 9 | 0 | 0.00  (0.00-0.06) | <5 | <5 | NR^a^ |
| **2016** | 11 | <5 | NR^a^ | <5 | 0 | 0.00  (0.00-0.17) | 5 | <5 | NR^a^ | 7 | 0 | 0.00  (0.00-0.02) |
| **2017** | 8 | 0 | 0.00  (0.00-0.02) | <5 | 0 | 0.00  (0.00-0.21) | <5 | 0 | 0.00  (0.00-0.11) | <5 | 0 | 0.00  (0.00-0.02) |
| **2018** | 7 | 6 | 0.03  (0.01-0.07) | 5 | 5 | 0.36  (0.12-0.85) | <5 | <5 | NR^a^ | 0 | 0 | 0.00  (0.00-0.03) |
| **2019** | <5 | <5 | NR^a^ | <5 | 0 | 0.00  (0.00-0.32) | 0 | 0 | 0.00  (0.00-0.15) | <5 | <5 | NR^a^ |
| **Trend test**  **(p-value)** | - | - | 0.170 | - | - | 0.507 | - | - | 0.005 | - | - | 0.028 |

^a^Not reported: Where the number of hospital admissions were less than 5 the data are not reported, in accordance with CPRD and HES-APC data protection policies. CI: confidence interval; GP: general practice; HCRU: healthcare resource utilisation; N: number; PP: pneumococcal pneumonia.

**S8 Table. ACP HCRU in hospital setting by study year**

|  | **All children** | | | **<2 years** | | | **2-4 years** | | | **5-17 years** | | |
| --- | --- | --- | --- | --- | --- | --- | --- | --- | --- | --- | --- | --- |
|  | **N of episodes** | **N of hospital admissions** | **Inpatient admission yearly rate per 1,000 patients**  **(95% CI)** | **N of episodes** | **N of hospital admissions** | **Inpatient admission yearly rate per 1,000 patients**  **(95% CI)** | **N of episodes** | **N of hospital admissions** | **Inpatient admission yearly rate per 1,000 patients**  **(95% CI)** | **N of episodes** | **N of hospital admissions** | **Inpatient admission yearly rate per 1,000 patients**  **(95% CI)** |
| **2003** | 719 | 518 | 1.10  (1.01-1.20) | 216 | 168 | 4.67  (3.99-5.43) | 279 | 204 | 2.64  (2.29-3.03) | 224 | 146 | 0.41  (0.34-0.48) |
| **2004** | 836 | 618 | 1.23  (1.14-1.33) | 288 | 243 | 5.68  (4.99-6.44) | 306 | 220 | 2.73  (2.38-3.12) | 242 | 155 | 0.41  (0.35-0.48) |
| **2005** | 945 | 715 | 1.37  (1.27-1.47) | 296 | 227 | 5.00  (4.37-5.69) | 326 | 250 | 2.97  (2.61-3.36) | 323 | 238 | 0.61  (0.53-0.69) |
| **2006** | 960 | 757 | 1.41  (1.31-1.51) | 273 | 222 | 4.73  (4.13-5.40) | 339 | 268 | 3.05  (2.69-3.44) | 348 | 267 | 0.66  (0.58-0.75) |
| **2007** | 894 | 689 | 1.25  (1.16-1.35) | 266 | 207 | 4.19  (3.64-4.80) | 334 | 249 | 2.73  (2.40-3.09) | 294 | 233 | 0.57  (0.50-0.65) |
| **2008** | 872 | 692 | 1.23  (1.14-1.33) | 292 | 232 | 4.53  (3.97-5.15) | 299 | 235 | 2.48  (2.17-2.81) | 281 | 225 | 0.54  (0.47-0.62) |
| **2009** | 1,487 | 693 | 1.23  (1.14-1.32) | 341 | 224 | 4.36  (3.81-4.97) | 439 | 238 | 2.46  (2.16-2.79) | 707 | 231 | 0.55  (0.48-0.63) |
| **2010** | 968 | 795 | 1.42  (1.32-1.52) | 286 | 221 | 4.35  (3.79-4.96) | 378 | 330 | 3.38  (3.03-3.77) | 304 | 244 | 0.59  (0.52-0.67) |
| **2011** | 920 | 778 | 1.43  (1.33-1.53) | 275 | 226 | 4.48  (3.92-5.11) | 340 | 304 | 3.18  (2.83-3.55) | 305 | 248 | 0.62  (0.55-0.70) |
| **2012** | 884 | 727 | 1.35  (1.25-1.45) | 256 | 210 | 4.22  (3.67-4.83) | 328 | 283 | 2.98  (2.64-3.35) | 300 | 234 | 0.59  (0.52-0.67) |
| **2013** | 656 | 543 | 1.07  (0.98-1.17) | 212 | 151 | 3.30  (2.79-3.87) | 242 | 219 | 2.47  (2.15-2.82) | 202 | 173 | 0.46  (0.40-0.54) |
| **2014** | 581 | 485 | 1.11  (1.01-1.21) | 174 | 141 | 3.76  (3.16-4.43) | 204 | 181 | 2.37  (2.03-2.74) | 203 | 163 | 0.50  (0.43-0.59) |
| **2015** | 418 | 351 | 1.00  (0.89-1.11) | 130 | 104 | 3.54  (2.89-4.29) | 157 | 129 | 2.13  (1.78-2.53) | 131 | 118 | 0.45  (0.37-0.54) |
| **2016** | 372 | 335 | 1.31  (1.17-1.46) | 99 | 75 | 3.52  (2.77-4.41) | 139 | 128 | 2.94  (2.45-3.50) | 134 | 132 | 0.69  (0.58-0.82) |
| **2017** | 236 | 188 | 0.91  (0.79-1.05) | 71 | 57 | 3.28  (2.48-4.25) | 83 | 60 | 1.76  (1.34-2.26) | 82 | 71 | 0.46  (0.36-0.58) |
| **2018** | 219 | 201 | 1.15  (1.00-1.32) | 62 | 57 | 4.13  (3.13-5.35) | 92 | 91 | 3.17  (2.55-3.89) | 65 | 53 | 0.40  (0.30-0.53) |
| **2019** | 175 | 158 | 1.07  (0.91-1.25) | 34 | 29 | 2.55  (1.70-3.66) | 73 | 80 | 3.36  (2.66-4.18) | 68 | 49 | 0.44  (0.32-0.58) |
| **Trend test**  **(p-value)** | - | - | 0.126 | - | - | <0.001 | - | - | 0.805 | - | - | 0.364 |

ACP: all-cause pneumonia; CI: confidence interval; GP: general practice; HCRU: healthcare resource utilisation; N: number.

**S9 Table. PP costs per episode by study year**

|  | **All children** | | **<2 years** | | **2-4 years** | | **5-17 years** | |
| --- | --- | --- | --- | --- | --- | --- | --- | --- |
|  | **Primary care cost per episode, £ (95% CI)** | **Inpatient cost per episode, £ (95% CI)** | **Primary care cost per episode, £ (95% CI)** | **Inpatient cost per episode, £ (95% CI)** | **Primary care cost per episode, £ (95% CI)** | **Inpatient cost per episode, £ (95% CI)** | **Primary care cost per episode, £ (95% CI)** | **Inpatient cost per episode, £ (95% CI)** |
| **2003** | 42.4  (38.8-46.0) | 438  (0.0-913) | 41.6  (39.0-44.2) | NR^a^ | 43.3  (36.3-50.3) | 743  (0.0-1,811) | 41.9  (35.5-48.4) | NR^a^ |
| **2004** | 39.6  (35.3-43.8) | 573  (177-970) | 35.1  (24.8-45.3) | 924  (26.6-1,821) | 39.6  (36.1-43.1) | 448  (0.0-1,099) | 43.3  (33.7-52.8) | 436  (0.0-1,082) |
| **2005** | 38.7  (34.8-42.5) | 1,619  (628-2,609) | 33.6  (26.9-40.4) | 2,640  (116-5,163) | 38.7  (31.4-45.9) | 2,128  (2.8-4,253) | 41.5  (35.4-47.5) | 553  (0.0-1,174) |
| **2006** | 39.5  (35.2-43.8) | 1,254  (494-2,014) | 37.6  (28.2-46.9) | 2,126  (0.0-4,667) | 37.1  (32.4-41.8) | 1,019  (0.0-2,164) | 42.5  (34.4-50.6) | 940  (193-1,688) |
| **2007** | 39.0  (34.2-43.9) | 1,881  (560-3,202) | 33.6  (25.6-41.6) | 1,463  (0.0-3,151) | 48.5  (32.5-64.5) | 1,056  (0.0-3,274) | 37.1  (32.2-41.9) | 2,447  (194-4,700) |
| **2008** | 34.5  (29.4-39.7) | 2,739  (652-4,827) | 36.1  (29.8-42.4) | 1,980  (0.0-5,070) | 31.9  (22.6-41.1) | 4,180  (0.0-10,044) | 35.6  (24.8-46.3) | 2,107  (385-3,828) |
| **2009** | 36.3  (31.1-41.5) | 1,850  (303-3,398) | 33.4  (23.2-43.6) | 1,184  (0.0-2,403) | 32.5  (22.5-42.5) | 3,832  (0.0-8,863) | 41.0  (32.7-49.3) | 903  (0.0-2,227) |
| **2010** | 34.4  (29.6-39.3) | 2,759  (1,242-4,275) | 39.0 (-)^b^ | NR^a^ | 38.5  (31.7-45.2) | 3,045  (0.0-6,138) | 29.8  (21.0-38.6) | 3,231  (1,072-5,391) |
| **2011** | 40.9  (35.9-45.8) | 899  (0.0-1,827) | 38.1  (33.0-43.1) | 627  (0.0-1,950) | 44.0  (28.8-59.3) | 1,596  (0.0-5,152) | 41.5  (32.8-50.1) | 784  (0.0-1,971) |
| **2012** | 41.2  (35.4-47.0) | 475  (0.0-1,017) | 32.5  (19.7-45.2) | 1,393  (0.0-3,518) | 46.4  (39.0-53.8) | 0.0 (-)^b^ | 41.9  (30.5-53.2) | NR^a^ |
| **2013** | 34.1  (27.9-40.3) | 1,596  (423-2,769) | 35.4  (15.8-55.0) | NR^a^ | 39.6  (31.9-47.3) | 752  (0.0-1,860) | 26.6  (14.1-39.2) | 3,030  (98.9-5,962) |
| **2014** | 35.7  (29.0-42.3) | 1,254  (204-2,304) | 31.3  (17.2-45.5) | 1,026.0  (0.0-2,375) | 33.4  (19.8-47.1) | 1,075  (0.0-3,705) | 42.0  (32.5-51.6) | 1,630  (0.0-4,098) |
| **2015** | 40.6  (32.7-48.6) | 2,341  (0.0-7,361) | NR^a^ | NR^a^ | 45.2  (36.7-53.8) | 0.0 (-)^b^ | NR^a^ | NR^a^ |
| **2016** | 49.7  (38.7-60.7) | NR^a^ | NR^a^ | NR^a^ | 45.3  (37.0-53.5) | NR^a^ | 56.2  (27.9-84.5) | NR^a^ |
| **2017** | 43.8  (38.1-49.4) | 0.0 (-)^b^ | NR^a^ | NR^a^ | NR^a^ | NR^a^ | NR^a^ | NR^a^ |
| **2018** | 27.9  (10.3-45.5) | 4,299  (0.0-11,123) | NR^a^ | 2,257  (0.0-7,695) | NR^a^ | NR^a^ | NR^a^ | NR^a^ |
| **2019** | NR^a^ | NR^a^ | NR^a^ | NR^a^ | NR^a^ | NR^a^ | NR^a^ | NR^a^ |
| **Trend test**  **(p-value)** | 0.753 | 0.400 | 0.169 | 0.592 | 0.546 | 0.714 | 0.854 | 0.640 |

^a^Not reported: Where the number of GP visits or hospital admissions were less than 5 the data are not reported, in accordance with CPRD and HES-APC data protection policies. ^b^95% CI not calculated when there is no variability in data. CI: confidence interval; N: number; PP: pneumococcal pneumonia.

**S10 Table. ACP costs per episode by study year**

|  | **All children** | | **<2 years** | | **2-4 years** | | **5-17 years** | |
| --- | --- | --- | --- | --- | --- | --- | --- | --- |
|  | **Primary care cost per episode, £ (95% CI)** | **Inpatient cost per episode, £ (95% CI)** | **Primary care cost per episode, £ (95% CI)** | **Inpatient cost per episode, £ (95% CI)** | **Primary care cost per episode, £ (95% CI)** | **Inpatient cost per episode, £ (95% CI)** | **Primary care cost per episode, £ (95% CI)** | **Inpatient cost per episode, £ (95% CI)** |
| **2003** | 32.8  (30.9-34.6) | 4,841  (2,244-7,439) | 29.9  (26.9-33.0) | 8,401  (0.0-17,012) | 30.4  (27.5-33.3) | 3,231  (2,819-3,643) | 38.4  (34.9-41.9) | 3,407  (2,641-4,173) |
| **2004** | 31.6  (30.0-33.2) | 3,247  (2,905-3,589) | 27.4  (24.6-30.1) | 4,031  (3,277-4,786) | 33.0  (30.6-35.4) | 2,927  (2,416-3,438) | 34.8  (31.6-38.1) | 2,719  (2,309-3,128) |
| **2005** | 32.6  (31.0-34.2) | 3,744  (3,403-4,086) | 28.5  (25.9-31.0) | 4,421  (3,725-5,117) | 33.3  (30.5-36.0) | 3,459  (2,890-4,028) | 35.6  (32.7-38.5) | 3,412  (2,900-3,925) |
| **2006** | 32.6  (31.0-34.3) | 3,888  (3,195-4,582) | 27.6  (24.6-30.7) | 4,079  (3,321-4,837) | 32.3  (29.8-34.8) | 3,135  (2,708-3,562) | 36.8  (34.0-39.6) | 4,469  (2,709-6,228) |
| **2007** | 30.4  (28.7-32.0) | 3,977  (3,507-4,446) | 24.0  (21.3-26.7) | 4,588  (3,384-5,792) | 32.0  (29.2-34.9) | 3,388  (2,883-3,893) | 34.3  (31.3-37.3) | 4,083  (3,366-4,800) |
| **2008** | 28.3  (26.7-29.9) | 4,202  (3,687-4,717) | 23.1  (20.7-25.6) | 4,702  (3,529-5,874) | 29.2  (26.6-31.8) | 4,014  (3,233-4,796) | 32.7  (29.5-35.9) | 3,884  (3,256-4,512) |
| **2009** | 33.3  (32.3-34.3) | 2,237  (1,972-2,502) | 27.0  (24.6-29.4) | 3,379  (2,756-4,002) | 33.0  (31.2-34.7) | 2,652  (2,029-3,275) | 36.6  (35.3-37.9) | 1,430  (1,175-1,684) |
| **2010** | 26.5  (25.0-28.0) | 4,231  (3,658-4,803) | 23.3  (20.8-25.9) | 4,486  (3,078-5,894) | 26.7  (24.3-29.1) | 3,947  (3,236-4,657) | 29.3  (26.6-32.1) | 4,335  (3,453-5,216) |
| **2011** | 26.7  (25.1-28.3) | 4,537  (3,847-5,227) | 23.9  (21.1-26.8) | 4,244  (3,320-5,168) | 25.1  (22.5-27.6) | 4,606  (3,359-5,853) | 31.0  (28.2-33.8) | 4,729  (3,405-6,053) |
| **2012** | 24.1  (22.6-25.7) | 4,099  (3,561-4,637) | 20.2  (17.5-22.8) | 4,141  (3,304-4,977) | 24.9  (22.2-27.5) | 4,115  (3,007-5,223) | 26.8  (24.0-29.5) | 4,046  (3,300-4,791) |
| **2013** | 25.3  (23.4-27.3) | 4,549  (3,643-5,454) | 22.7  (19.7-25.8) | 4,329  (2,726-5,932) | 24.5  (21.4-27.5) | 4,370  (2,707-6,034) | 29.1  (25.2-33.0) | 4,994  (3,601-6,386) |
| **2014** | 23.1  (21.1-25.1) | 3,627  (3,089-4,165) | 23.6  (20.2-27.1) | 3,299  (2,474-4,124) | 21.3  (18.1-24.5) | 3,622  (2,838-4,405) | 24.5  (20.9-28.1) | 3,917  (2,784-5,050) |
| **2015** | 24.0  (21.7-26.3) | 4,130  (3,448-4,812) | 25.4  (21.5-29.3) | 3,786  (2,823-4,750) | 24.1  (19.9-28.3) | 2,998  (2,397-3,599) | 22.5  (18.7-26.4) | 5,870  (4,044-7,696) |
| **2016** | 20.6  (18.2-23.1) | 3,723  (3,120-4,325) | 19.9  (15.5-24.3) | 2,738  (1,969-3,508) | 19.5  (15.9-23.1) | 2,858  (2,405-3,311) | 22.3  (17.7-26.9) | 5,332  (3,863-6,800) |
| **2017** | 21.7  (18.7-24.6) | 3,390  (2,683-4,096) | 18.6  (13.3-23.9) | 2,935  (1,966-3,904) | 21.2  (16.4-26.0) | 3,940  (2,256-5,624) | 24.8  (19.3-30.3) | 3,222  (2,449-3,996) |
| **2018** | 18.4  (15.5-21.3) | 3,918  (2,997-4,839) | 16.6  (11.5-21.7) | 4,025  (3,000-5,050) | 14.8  (10.3-19.2) | 3,339  (2,706-3,972) | 25.4  (19.9-30.9) | 4,658  (1,741-7,574) |
| **2019** | 19.0  (15.4-22.6) | 4,032  (3,071-4,993) | 16.1  (8.7-23.6) | 4,315  (1,861-6,769) | 15.7  (10.7-20.6) | 4,345  (2,627-6,064) | 24.1  (17.4-30.7) | 3,543  (2,356-4,731) |
| **Trend test**  **(p-value)** | <0.001 | 0.967 | <0.001 | 0.036 | <0.001 | 0.343 | <0.001 | 0.127 |

ACP: all-cause pneumonia; CI: confidence interval; N: number.

**S11 Table. AOM HCRU by study year**

|  | **All children** | | | **<2 years** | | | **2-4 years** | | | **5-17 years** | | |
| --- | --- | --- | --- | --- | --- | --- | --- | --- | --- | --- | --- | --- |
|  | **N of episodes** | **N of GP visits** | **GP visits yearly rate per 1,000 patients**  **(95% CI)** | **N of episodes** | **N of GP visits** | **GP visits yearly rate per 1,000 patients**  **(95% CI)** | **N of episodes** | **N of GP visits** | **GP visits yearly rate per 1,000 patients**  **(95% CI)** | **N of episodes** | **N of GP visits** | **GP visits yearly rate per 1,000 patients**  **(95% CI)** |
| **2003** | 22,899 | 23,969 | 50.89  (50.25-51.54) | 3,799 | 4,068 | 113.01  (109.56-116.54) | 8,235 | 8,639 | 111.81  (109.46-114.19) | 10,865 | 11,262 | 31.48  (30.90-32.07) |
| **2004** | 21,629 | 22,666 | 45.17  (44.59-45.76) | 4,425 | 4,749 | 111.03  (107.90-114.24) | 7,425 | 7,757 | 96.32  (94.19-98.49) | 9,779 | 10,160 | 26.85  (26.33-27.37) |
| **2005** | 21,910 | 22,963 | 43.96  (43.39-44.53) | 4,104 | 4,387 | 96.60  (93.76-99.50) | 7,674 | 8,026 | 95.41  (93.33-97.52) | 10,132 | 10,550 | 26.86  (26.35-27.37) |
| **2006** | 21,375 | 22,383 | 41.59  (41.04-42.14) | 4,059 | 4,323 | 92.19  (89.46-94.98) | 7,713 | 8,087 | 91.99  (90.00-94.02) | 9,603 | 9,973 | 24.72  (24.24-25.21) |
| **2007** | 21,813 | 22,767 | 41.34  (40.81-41.88) | 4,211 | 4,495 | 90.98  (88.34-93.68) | 7,882 | 8,208 | 89.92  (87.98-91.88) | 9,720 | 10,064 | 24.54  (24.07-25.03) |
| **2008** | 22,175 | 23,206 | 41.30  (40.77-41.83) | 4,605 | 4,877 | 95.24  (92.59-97.96) | 7,966 | 8,337 | 87.86  (85.98-89.76) | 9,604 | 9,992 | 24.03  (23.56-24.51) |
| **2009** | 20,999 | 21,881 | 38.70  (38.19-39.22) | 4,443 | 4,709 | 91.63  (89.03-94.29) | 7,459 | 7,755 | 80.18  (78.41-81.98) | 9,097 | 9,417 | 22.57  (22.12-23.03) |
| **2010** | 20,894 | 21,765 | 38.92  (38.41-39.44) | 4,219 | 4,460 | 87.75  (85.19-90.36) | 7,756 | 8,067 | 82.67  (80.87-84.49) | 8,919 | 9,238 | 22.49  (22.03-22.95) |
| **2011** | 19,098 | 19,897 | 36.46  (35.96-36.97) | 3,891 | 4,062 | 80.54  (78.08-83.06) | 6,942 | 7,260 | 75.85  (74.12-77.62) | 8,265 | 8,575 | 21.46  (21.01-21.92) |
| **2012** | 20,639 | 21,494 | 39.89  (39.36-40.42) | 4,019 | 4,260 | 85.58  (83.03-88.19) | 7,677 | 7,972 | 83.89  (82.06-85.75) | 8,943 | 9,262 | 23.50  (23.03-23.99) |
| **2013** | 16,626 | 17,301 | 34.15  (33.64-34.66) | 3,333 | 3,513 | 76.77  (74.25-79.35) | 6,263 | 6,511 | 73.32  (71.55-75.13) | 7,030 | 7,277 | 19.56  (19.11-20.01) |
| **2014** | 13,764 | 14,290 | 32.71  (32.17-33.25) | 2,590 | 2,728 | 72.66  (69.96-75.44) | 5,261 | 5,479 | 71.60  (69.72-73.52) | 5,913 | 6,083 | 18.84  (18.37-19.32) |
| **2015** | 10,315 | 10,778 | 30.58  (30.00-31.16) | 2,079 | 2,206 | 75.09  (71.99-78.29) | 3,857 | 4,012 | 66.20  (64.16-68.28) | 4,379 | 4,560 | 17.37  (16.87-17.88) |
| **2016** | 7,113 | 7,399 | 28.89  (28.24-29.56) | 1,289 | 1,359 | 63.72  (60.38-67.20) | 2,724 | 2,825 | 64.89  (62.52-67.33) | 3,100 | 3,215 | 16.81  (16.24-17.40) |
| **2017** | 5,026 | 5,243 | 25.43  (24.75-26.13) | 1,092 | 1,156 | 66.52  (62.74-70.46) | 1,821 | 1,906 | 55.77  (53.29-58.33) | 2,113 | 2,181 | 14.11  (13.52-14.71) |
| **2018** | 4,445 | 4,618 | 26.45  (25.70-27.23) | 869 | 925 | 67.04  (62.79-71.50) | 1,709 | 1,760 | 61.24  (58.41-64.17) | 1,867 | 1,933 | 14.64  (13.99-15.31) |
| **2019** | 3,288 | 3,423 | 23.22  (22.45-24.01) | 710 | 749 | 65.73  (61.11-70.61) | 1,277 | 1,332 | 55.90  (52.94-58.98) | 1,301 | 1,342 | 11.96  (11.33-12.62) |
| **Trend test**  **(p-value)** | - | - | <0.001 | - | - | <0.001 | - | - | <0.001 | - | - | <0.001 |

AOM: acute otitis media; CI: confidence interval; GP: general practice; HCRU: healthcare resource utilisation; N: number.

**S12 Table. AOM costs per episode by study year**

|  | **Primary care cost per episode, £ (95% CI)** | | | |
| --- | --- | --- | --- | --- |
|  | **All children** | **<2 years** | **2-4 years** | **5-17 years** |
| **2003** | 48.4 (48.3-48.6) | 49.0 (48.5-49.5) | 48.1 (47.8-48.3) | 48.5 (48.3-48.7) |
| **2004** | 48.4 (48.3-48.6) | 49.0 (48.6-49.4) | 47.8 (47.6-48.0) | 48.6 (48.4-48.8) |
| **2005** | 48.5 (48.3-48.7) | 49.0 (48.6-49.4) | 47.8 (47.5-48.0) | 48.8 (48.6-49.1) |
| **2006** | 48.5 (48.3-48.6) | 48.8 (48.4-49.2) | 48.0 (47.8-48.3) | 48.7 (48.5-49.0) |
| **2007** | 48.4 (48.3-48.6) | 48.8 (48.4-49.2) | 47.8 (47.6-48.0) | 48.7 (48.5-49.0) |
| **2008** | 48.7 (48.5-48.8) | 48.6 (48.3-49.0) | 48.1 (47.9-48.4) | 49.1 (48.9-49.3) |
| **2009** | 48.5 (48.4-48.7) | 48.6 (48.2-48.9) | 47.9 (47.7-48.2) | 49.0 (48.8-49.3) |
| **2010** | 48.8 (48.6-48.9) | 48.7 (48.3-49.0) | 47.9 (47.7-48.2) | 49.5 (49.2-49.7) |
| **2011** | 48.8 (48.6-49.0) | 48.1 (47.8-48.4) | 48.1 (47.9-48.4) | 49.6 (49.4-49.9) |
| **2012** | 49.0 (48.9-49.2) | 48.7 (48.3-49.1) | 48.2 (47.9-48.4) | 49.9 (49.6-50.1) |
| **2013** | 49.0 (48.9-49.2) | 48.6 (48.1-49.0) | 48.2 (47.9-48.4) | 50.0 (49.7-50.3) |
| **2014** | 49.5 (49.3-49.6) | 48.8 (48.3-49.2) | 48.9 (48.6-49.2) | 50.2 (49.9-50.5) |
| **2015** | 50.8 (50.5-51.0) | 49.6 (49.0-50.1) | 50.6 (50.3-51.0) | 51.4 (51.0-51.8) |
| **2016** | 51.3 (51.0-51.6) | 49.5 (48.9-50.1) | 51.7 (51.2-52.1) | 51.7 (51.2-52.1) |
| **2017** | 52.1 (51.8-52.5) | 50.8 (50.0-51.6) | 53.3 (52.7-53.9) | 51.7 (51.2-52.2) |
| **2018** | 52.3 (51.9-52.6) | 51.1 (50.2-52.1) | 52.9 (52.4-53.4) | 52.3 (51.8-52.8) |
| **2019** | 52.3 (51.8-52.7) | 50.6 (49.6-51.6) | 54.0 (53.3-54.7) | 51.5 (50.8-52.1) |
| **Trend test**  **(p-value)** | <0.001 | 0.197 | <0.001 | <0.001 |

AOM: acute otitis media; CI: confidence interval; N: number.
